# Response: Use of integrated genomic surveillance by local public health authorities: Recommendations based on a mixed-methods study of current adoption, applications and success factors, Germany, 2023

**DOI:** 10.2807/1560-7917.ES.2025.30.28.2500508

**Published:** 2025-07-17

**Authors:** Anna Bludau, Johannes Dreesman, Richard Egelkamp, Lutz Ehlkes, Fabian Feil, Hajo Grundmann, Masyar Monazahian, Markus Tröger, Alexander Dilthey, Claudia Hornberg, Simone Scheithauer

**Affiliations:** 1 Department of Infection Control and Infectious Diseases, University Medical Center Göttingen, Georg August University Göttingen, Göttingen, Germany; 2 Public Health Agency of Lower Saxony, Hanover, Germany; 3 Düsseldorf Health Authority (Gesundheitsamt Düsseldorf), Düsseldorf, Germany; 4 Institute for Infection Prevention and Control, Medical Center - University of Freiburg, Freiburg, Germany; 5 Bielefeld University, Medical School OWL, Sustainable Environmental Health Sciences, Bielefeld, Germany; 6 Institute of Medical Microbiology and Hospital Hygiene, University Hospital Düsseldorf, Heinrich-Heine-University Düsseldorf, Düsseldorf, Germany

To the editor: We thank the authors for their comments on our recently published article ‘*Use of integrated genomic surveillance by local public health authorities: Recommendations based on a mixed-methods study of current adoption, applications and success factors, Germany, 2023*’ [1].

We would like to emphasise that we agree with the key points raised. In particular, we fully acknowledge the central role of federal public health authorities in coordinating and implementing genomic pathogen surveillance efforts, including the prioritisation of public health-relevant pathogens for genomic analysis. We have already been committed to further develop our shared concept [2,3] with the federal public health authority in Germany, the Robert Koch Institute, to align efforts, avoid fragmentation and jointly strengthen the German genomic pathogen surveillance infrastructure.

Specifically, federal-based structures should not be replaced or duplicated. Instead, we emphasise the importance of integrating complementary expertise, infrastructure, and data access that can support public health authorities on all levels. The efficient implementation of genomic surveillance requires central coordination as well as bottom-up initiatives which, when structurally organised in the form of hierarchically distributed networks (such as in [4]), may specifically contribute to resilience and efficiency. One particular challenge concerns the establishment of suitable infrastructures for data exchange between all relevant parties, including public health authorities of all levels and academic research institutions.

We appreciate the ongoing dialogue on this important topic and look forward to continued collaboration with national and state level as well as local public health authorities towards creating one coordinated and resilient infrastructure for genomic pathogen surveillance in Germany. In addition, we would welcome the comparison and evaluation of different nationally adapted concepts within Europe and beyond, and if necessary, the joint development of these concepts.
